# Rare Case of Dental Implantation in the Segment with Residual Filling Material in the Mandibular Canal

**DOI:** 10.1155/2020/2689353

**Published:** 2020-07-23

**Authors:** Yury Georgievich Sedov, Kamil Nail'evich Khabiev, Zulfiya Iltuzurovna Yarulina, Vasiliy Stanislavovich Tarasuk, Anatoliy Mikhailovich Avanesov, Nikolay Ivanovich Sergeev, Vitaliy Georgievich Pantsulaya, Irina Gennad'evna Sedova

**Affiliations:** ^1^Department of General and Clinical Dentistry, RUDN University, Medical Institute, Moscow, Russia; ^2^Private Dental Practice, Moscow, Russia; ^3^Department of Orthopedic Dentistry, Kazan State Medical University of the Ministry of Health of Russia, Kazan, Russia; ^4^Scientific Centre of Roentgenoradiology and Russian National Research Medical University, n.a. N.I. Pirogov, Moscow, Russia

## Abstract

Dental implantation is the most popular method of restoring lost teeth. There are risk factors for dental implantation. These risk factors include the localization of residual filling material in the lumen of the mandibular canal in the selected jaw segment for implantation. A rare clinical case of dental implant placement with preservation of the safety zone relative to the residual siler in the mandibular canal is presented. A surgical guide was used for precise positioning. The treatment protocol was carried out without an immediate loading stage to monitor the possible development of symptoms.

## 1. Introduction

Dental implantation is the most popular method of lost teeth restoration [1, 2]. The complications of dental implants vary in the range of 5-10% [3, 4]. An important component of this method of treatment is the planning stage and, in particular, the use of radiological examination methods [5, 6]. To date, we recommend the use of cone-beam computed tomography, which allows the doctor to determine if there is enough bone volume for implant placement and the type of architectonic bone and also to take into account the location of important anatomical structures. On the lower jaw, such a structure includes the mandibular channel. Its diameter is 2-3 mm on average, and it is important for the clinician to know that in 80% of cases, the vessels are located in the upper part of the channel, and the nerve is located below them [7, 8]. Another anatomical feature is that the lower alveolar nerve is the third branch of the trigeminal nerve; it means that it is a large formation and has polyphasticity, which together increases its regenerative abilities [9].

Existing recommendations dictate that in order to avoid nerve damage, it is recommended to observe a safety zone of 2 mm from the apex of the implant to the upper border of the mandibular channel [10]. However, if there is a violation of the integrity of the channel, there are currently no clear official clinical recommendations on how to rehabilitate such a patient. The situation may get worse if you need to install a dental implant and there is already a residual filling material in the interesting area, which is also visualized in the mandibular channel. According to the Seddona classification, there are three types of nerve damage: neuropraxia, axonotmesis, and neurotmesis. If the last two are characterized by structural damage to the nerve, then neuropraxia is a compression or stretching of the nerve [11]. Localization of the siler in the channel usually results in neuropraxia, and it is assumed that sensitivity can be restored if there is no structural damage to the epinephrium and, as a result, toxic effects on the axons [9].

In connection with aforesaid, the question arises whether it is possible to perform dental implantation in patients with available data on the localization of the filling material in the mandibular channel in the same jaw segment. As a demonstration and subsequent discussion, we want to cite a clinical case.

## 2. Clinical Case

**Figure 1:**
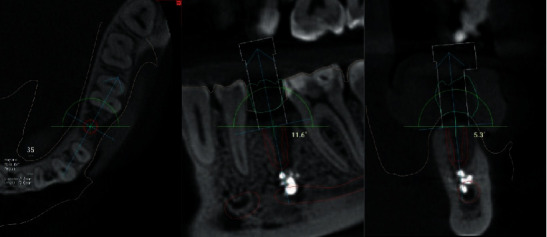
CBCT. The implant is installed virtually in the area of the missing 3.5 tooth. Fragments of filling material are visualized in the lumen of the mandibular canal.

The patient, 29 years old, went to the clinic to make a fixed structure in the area of the missing 3.5 tooth. The tooth was removed due to complications from apical periodontitis. During the consultation, the patient made panoramic zoning of the jaws, which showed a foreign body in this jaw segment projected into the mandibular channel. When collecting anamnesis, the patient denied any symptoms associated with damage to the inferior lunatic nerve. In view of the complexity of the situation and a more detailed analysis of the possibility of implantation, the patient was made cone-beam computed tomography with a special X-ray contrast positioner, so that in the future it will be possible to make a navigation guide. Analysis of the tomogram showed the presence of a foreign body, which was visualized as a heterogeneous, high-contrast round shadow with a clear, irregular contour of 4 × 3.4 mm in size, partially localized in the lumen of the mandibular channel, classified as a sealer. The foreign body was surrounded by bone tissue on all sides except the mandibular canal. The absence of symptoms is explained by the preservation of the integrity of the nerve sheath. In view of the complexity of the situation, it was finally agreed to conduct a dental implant with a navigation guide R2Gate to preserve the security zone between the apex of the implant and the top edge sealer, to avoid possibility of displacement in the channel. The entire planning protocol was conducted in digital mode (Figure 1).

Then, it was necessary to choose an implantation system. The best option in this case was the use of a so-called “supercortical” implant, which has a taper and a microthread on the neck. This makes it possible to achieve good primary stability in medium-density bone tissue and to ensure that the implant does not move deeper than planned [12].

Then, using 3D printing, a surgical guide was made for the full drilling protocol.

**Figure 2:**
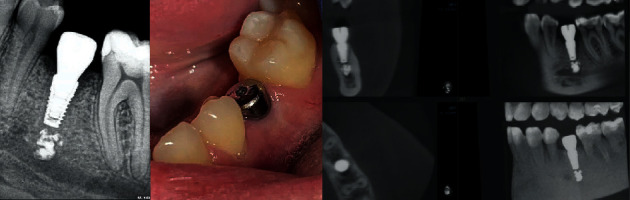
Intraoral X-ray. Condition in the oral cavity with the installation of the gum shaper. CBCT 1 month after implant placement.

The operation algorithm included anesthesia, positioning of the surgical guide on adjacent teeth, and a complete protocol for drilling through the guide with the installation of a dental implant. The drilling speed did not exceed 300 rpm. The torc when fixing the implant was 35 N/cm. The ISQ Index was equal to 69 units of CI. Since the use of a “supercortical” implant virtually eliminated the loss of stability at the obtained torc and ISQ values, a gum shaper with a diameter of 5.5 mm and a height of 5 mm was immediately installed. It is important to note that due to the complexity of the situation, it was decided to install a gingival cuff shaper for the observation period and not to carry out immediate loading, despite the design of the implant. The postoperative image showed the optimal location of the implant with the preservation of the safety zone between the apex and the sealer border (Figure 2).

**Figure 3:**
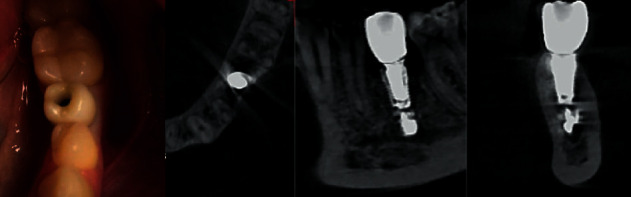
Fixing a permanent structure. CBCT. After the final restoration, the implant is also located in compliance with the safety zone.

During the three months of follow-up, the patient made no complaints. Before the prosthetics stage, the frequency-resonance analysis indicators demonstrated the onset of osteointegration. Prosthetics was performed with an all-zirconium crown on a titanium base with transocclusal fixation. Two months after fixation of the permanent crown, a CT scan was performed to assess the position of the implant after loading. According to CBCT, the distance from the implant apex to the upper border of the sealer is 0.72 mm. The peri-implant bone structure was normal. The patient did not complain (Figure 3).

## 3. Discussion

Injury to the lower alveolar nerve is a serious mistake in dental treatment. As a rule, this occurs after endodontic treatment and dental implantation [13]. The recommendations of most experts suggest surgical intervention for 72 hours in the presence of pain symptoms and determination of the mandibular channel damage on X-ray. In the absence of symptoms or its weak variations, the patient is undergoing dynamic observation or conservative therapy [14]. Filling material without damage to the nerve sheath may not cause clinical symptoms, while radiographs determined it in the lumen of the mandibular canal [9].

Based on our clinical case, it follows that restoration of the lost tooth in patients with diagnosed involvement of the mandibular canal due to dental treatment is possible. However, a preliminary assessment is required that combines the absence or presence of complaints with a CT scan analysis. If symptoms are present and damage to the integrity of the canal is visualized on the X-ray image, it is necessary to treat such a patient in the maxillofacial department. If the tomogram shows the presence of a foreign body in the lumen of the canal, but there are no symptoms, it is necessary to determine the identity of this foreign body and plan dental implantation. In our opinion, if the sealer is visualized, it is better not to carry out immediate loading even with adequate primary stability. This is due to the fact that before the onset of osseointegration, there may be excessive external pressure that can displace the implant, and this will aggravate the impact of the sealer on the n.h. channel. The safety zone was chosen from the apex of the implant to the sealer in 1 mm, because the distance to the channel exceeded 2 mm and even the tip of the cutter could not damage the integrity of this structure. Drilling speed up to 300 rpm helped to enhance the tactile sensation of the attending physician in the drilling process and reduce the risk of overheating the bone bed due to lack of irrigation due to the use of a surgical guide. The latter was the determining factor for controlling the location of the implant according to the virtual planning protocol.

## 4. Conclusion

The use of modern digital technologies allows for the transfer of this data intraoperatively. Even complex cases involving the mandibular canal due to iatrogenic treatment are not a contraindication to dental implantation but should be solved in each case individually from the analysis of symptoms and X-ray examination data. Also, it is recommended that the load on the implant is at the onset of osseointegration.

## Additional Points

*Materials and Tools Used*. The materials and tools used are implantation system Impro (ImproManagement, Germany), R2gate program (Megagen), and device for determining the stability of ISQ Penguin.
